# A Genomics England haplotype reference panel and imputation of UK Biobank

**DOI:** 10.1038/s41588-024-01868-7

**Published:** 2024-08-12

**Authors:** Sinan Shi, Simone Rubinacci, Sile Hu, Loukas Moutsianas, Alex Stuckey, Anna C. Need, Pier Francesco Palamara, Mark Caulfield, Jonathan Marchini, Simon Myers

**Affiliations:** 1 Department of Statistics, University of Oxford, Oxford, UK; 2 Harvard Medical School, Harvard University, Boston, MA USA; 3 Novo Nordisk Research Centre, Oxford, UK; 4 Genomics England, London, UK; 5 Queen Mary University of London, London, UK; 6 Regeneron Genetic Center, Tarrytown, NY USA

**Keywords:** Genetics research, Genome-wide association studies

## Abstract

We built a reference panel with 342 million autosomal variants using 78,195 individuals from the Genomics England (GEL) dataset, achieving a phasing switch error rate of 0.18% for European samples and imputation quality of *r*^2^ = 0.75 for variants with minor allele frequencies as low as 2 × 10^−4^ in white British samples. The GEL-imputed UK Biobank genome-wide association analysis identified 70% of associations found by direct exome sequencing (*P* < 2.18 × 10^−11^), while extending testing of rare variants to the entire genome. Coding variants dominated the rare-variant genome-wide association results, implying less disruptive effects of rare non-coding variants.

## Abstract (web summary)

A Genomics England haplotype reference panel constructed using sequence data from 78,195 individuals improves phasing and imputation accuracy. Imputation of the UK Biobank using this panel enables genome-wide rare-variant association analyses.

## Main

A key step in genome-wide association studies (GWAS) is imputation of untyped variants from those genotyped using a reference panel, allowing downstream testing of imputed sites. Reference panel quality substantially impacts results, particularly for low-frequency variants. Here, we build a reference panel with improved accuracy compared to existing panels using the Genomics England (GEL) high-coverage sequencing (30×) dataset, among the largest genetic variation resources yet collected in the United Kingdom^1^. We impute the UK Biobank samples across the whole genome and find several new rare-variant associations for tested traits. In our genome-wide analyses, high-confidence associations with large effect sizes only rarely occur away from coding sequences, suggesting that, although the most of the genome is non-coding, non-coding variants only occasionally possess effect sizes comparable to those of the strongest coding variants.

**Fig. 1:**
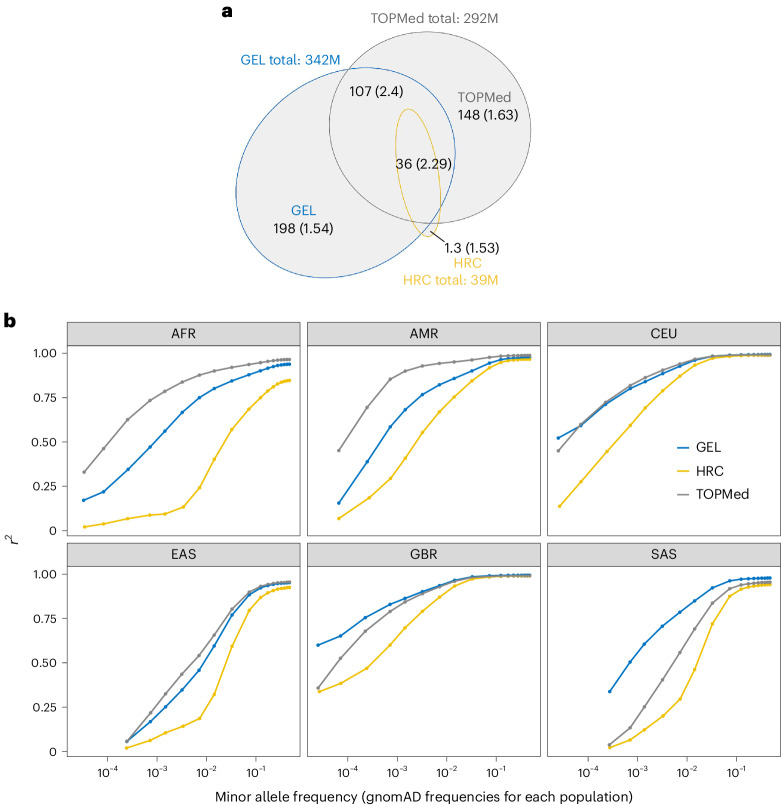
The GEL reference panel variant count and imputation accuracy. **a**, Venn diagram comparing numbers of variants from the GEL, HRC and TOPMed r2 reference panels. The numbers show the variant count (in millions of variants, M), followed by the Ts/Tv ratio of these variants in brackets. **b**, Imputation performance, measured by *r*^2^ (Methods), for imputation of 1000 Genomes Project samples with African (AFR), American (AMR), East Asian (EAS), British (GBR), North European (CEU) and South Asian (SAS) populations, using three different reference panels (labels). The variants are stratified by gnomAD allele frequency (v.3.3.1)^4^ of their corresponding population.

**Extended Data Fig. 1:**
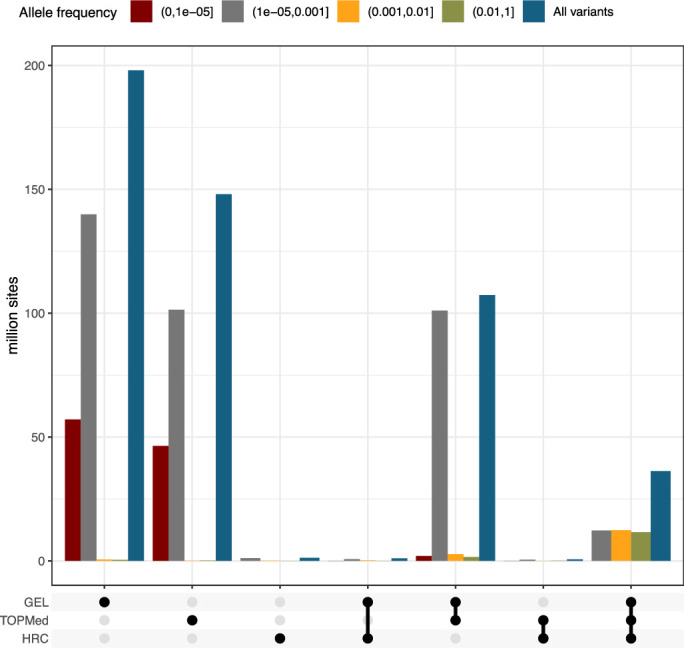
GEL, HRC and TOPMed reference panels variant counts. Allele frequency (colors) for variants existing in more than one reference panel is assigned to the highest allele frequency among all the panels.

The GEL study design intentionally samples many closely related individuals. This enhances the power of filters, including the Mendelian error filter, to eliminate false-positive calls and also allows more accurate phasing and imputation of rare variants. In particular, even variants found in only one or two individuals may be phased through transmission. The resulting GEL reference panel consists of 341,922,205 autosomal variants, with 31,502,703 (9.26%) being indels. Most detected variants are rare: 287.2 million (84.1%) have an allele frequency <0.0001, including 66.7 million (19.5%) singletons and 91.1 million (26.7%) doubletons. We compared GEL to the widely used TOPMed r2 (ref. ^2^) (we note that the r3 version containing ~30% more variants and samples was released while this manuscript was in preparation) and HRC^3^ panels, and found that GEL has 8 times and 1.1 times more variants than HRC and TOPMed, respectively (Fig. 1a and Extended Data Fig. 1). Owing to the use of mostly low-coverage sequencing technology, HRC has limited numbers of rare variants, especially those with allele frequency (AF) ≤ 10^−4^. While the numbers of rare variants captured in TOPMed and GEL are similar, around half of the ultra-rare variants (AF ≤ 10^−4^) from GEL and TOPMed are non-shared across the panels. As expected, all three panels capture a similar set of more common (AF > 10^−2^) variants, with <4% unique to each panel (Extended Data Fig. 1), indicating that common variants are largely saturated.

**Extended Data Fig. 2:**
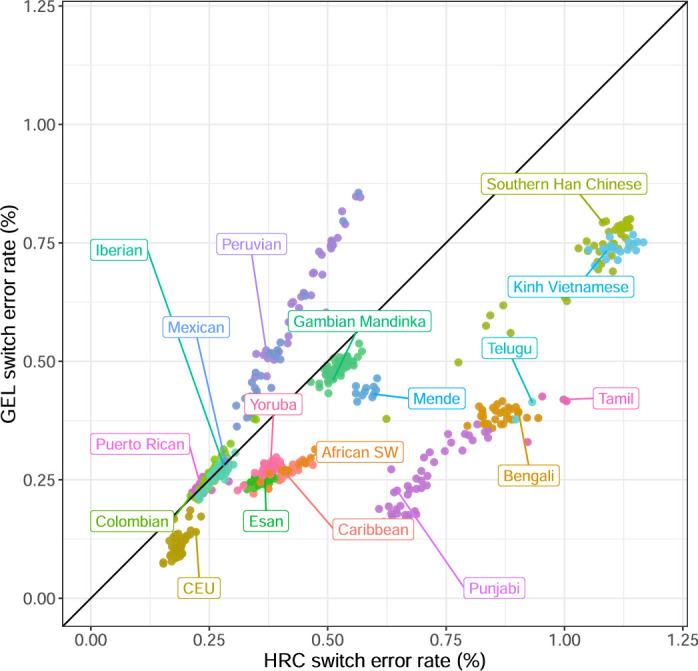
1000 Genome Project samples phasing switch error rate using GEL and HRC reference panels. Phasing accuracy for 589 high coverage 1000 Genome Project children from mother-father-child trio families, using HRC and GEL reference panels. The average GEL phasing switch error rates are 0.18%, 0.33%, 0.31% and 0.73% for European, African, South Asian, and East Asian samples, respectively.

The GEL reference panel provides a powerful resource for phasing European and South Asian samples due to their strong representation in the dataset. We compared phasing accuracy using the GEL and HRC reference panels on 1000 Genomes (1000 G) Project samples (Methods). GEL-based phasing achieved lower switch error rates than HRC phasing across 1000 G populations sampled from most worldwide regions (Extended Data Fig. 2), with HRC only showing improved performance for South American samples, which are largely absent from GEL.

**Extended Data Fig. 3:**
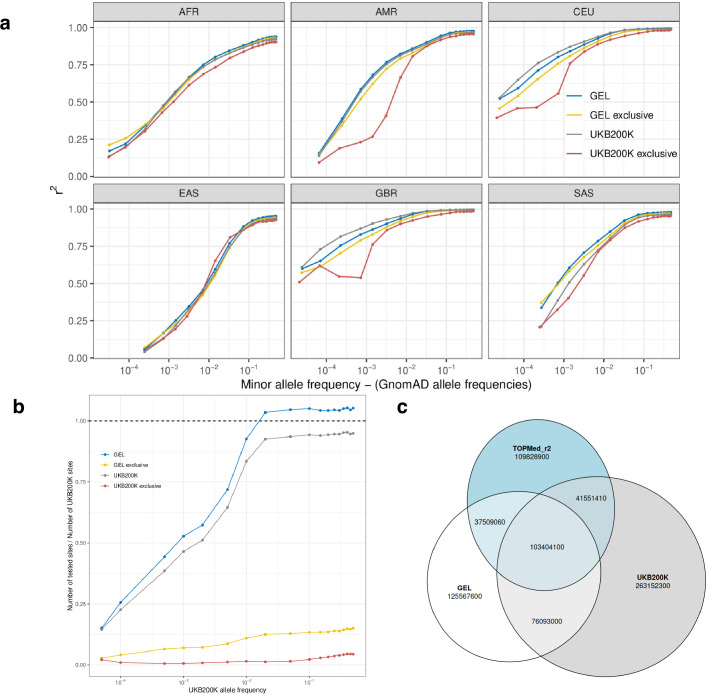
Imputation accuracy and site counts of GEL, TOPMed and UKB200K. **a**, 1000 Genome Project SNP imputation accuracy measured as the *r*^2^ between imputed dosages and the ground truth genotypes. ‘GEL’ and ‘UKB200K’ use all the SNPs from the respective reference panels, and ‘GEL exclusive’ and ‘UKB200K exclusive’ use only the SNPs that are not present in the other reference panel. **b**, The number of variants that are in overlap with 1000 G GBR samples with respect to the total number of SNPs of UKB200K. **c**, Venn diagram shows the autosomal site overlapping situation of GEL, TOPMed_r2 and UKB200K data, and the numbers indicate the variant count in each category.

**Extended Data Fig. 4:**
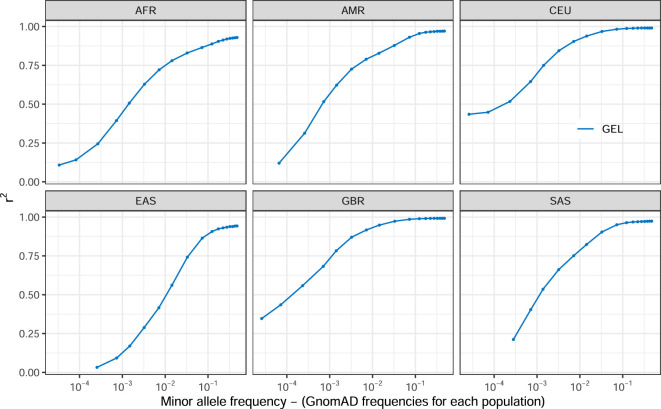
GEL indel imputation accuracy. GEL indel imputation accuracy of 1000 Genome Project samples, measured as the r^2^ between imputed dosages and the ground truth genotypes, stratified by 1000 Genome Project populations.

A primary use of the GEL resource will be as a reference panel for genotype imputation of other datasets. We assessed (Methods) the accuracy of imputation of 1000 G samples (from UKB single nucleotide polymorphism (SNP) array sites) using the GEL, TOPMed and HRC reference panels. Squared correlation *r*^2^ between the imputed allele dosages and true genotypes were calculated, stratified by the independently estimated genome aggregation database (gnomAD) (v.3.3.1) minor allele frequency (MAF)^4^. GEL achieved higher imputation *r*^2^ than HRC in all allele frequency bins for all ancestry groups and outperforms the TOPMed panel in white British (GBR) and South Asian (SAS) samples, especially for rarer variants: at MAF < 10^−5^, the GEL imputation *r*^2^ for GBR samples is 0.6, compared to 0.3 and 0.29 using TOPMed and HRC, respectively (Fig. 1b). The TOPMed panel outperforms GEL in African (AFR), American (AMR) and East Asian (EAS) samples due to its better representation from these groups (Fig. 1b). Examining imputation accuracy using the phased UKB 200 K high-coverage sequencing data as a reference panel^5^ (Supplementary Note and Extended Data Fig. 3) suggested substantial complementarity with GEL: similar overall imputation quality at the rarest variants with MAF ~10^−5^, slightly better imputation using UKB 200 K for shared MAF ~10^−4^–10^−2^ variants but more false-positive and false-negative variants for UKB 200 K compared to GEL. The GEL reference panel also imputed indels well: *r*^2^ = 0.74 at MAF = 10^−3^ for GBR samples (Extended Data Fig. 4).

**Extended Data Fig. 5:**
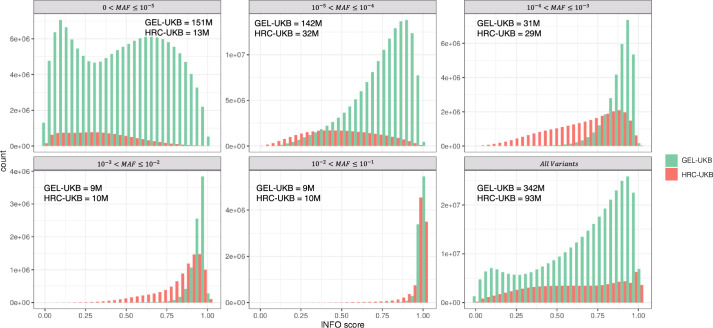
Imputation INFO score histogram comparison between GEL-UKB and HRC-UKB. Each panel shows the distribution of INFO scores for GEL and HRCUK10K imputed UK Biobank variants in different MAF bins. The total number of variants in each bin is provided in the panel legend.

**Extended Data Fig. 6:**
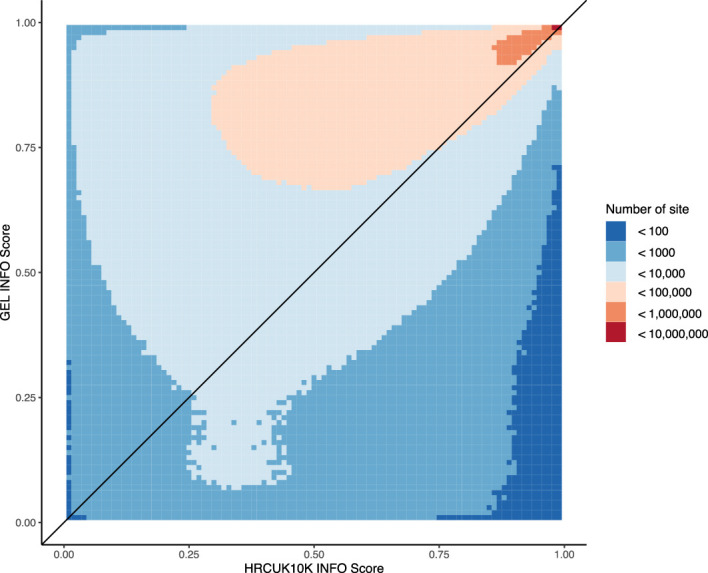
A heatmap of imputed UK Biobank INFO scores from the 65 million sites present in both GEL-UKB and HRC-UKB. GEL imputation of the UK Biobank (GEL-UKB) shows improved INFO scores for 87% of existing imputed markers in HRC-UKB. The *x*-axis shows imputation using HRC-UKB, and the *y*-axis shows imputation using GEL-UKB.

**Extended Data Fig. 7:**
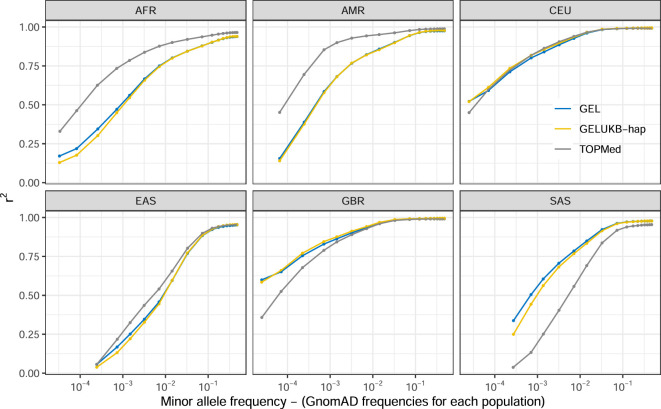
GELUKB-hap imputation accuracy. SNP imputation accuracy of 1000 Genome Project samples, measured as the *r*^2^ between imputed dosages and the ground truth genotypes, stratified by 1000 Genome Project populations, showing the performance of using GEL-UKB imputed haplotypes (GELUKB-hap) as reference panel compared to GEL and TOPMed overall SNP imputation accuracy.

We used the GEL panel to impute 488,315 UK Biobank samples at 342,573,817 variants, producing a ‘GEL-UKB’ dataset. Compared with the corresponding HRC and UK10K-imputed ‘HRC-UKB’^6^, GEL-UKB has around 3 times more variants, 3.5 times more missense variants and 6.6 times more ‘high impact consequence’ variants (Supplementary Table 1). The imputed information scores (Methods) were higher for GEL-UKB than HRC-UKB for 87% of variants they share, while 98% (78%) of GEL-imputed variants in the frequency range 10^−5^–10^−4^ (10^−6^–10^−5^) exceeded a threshold of 0.3 versus 78% (54%) for HRC (Extended Data Figs. 5 and 6). Finally, we tested the imputation potential from using the imputed GEL-UKB haplotypes (GELUKB-hap) as a reference panel in place of GEL itself. Again imputing 1000 G samples, we observed near-identical results (Extended Data Fig. 7) using GELUKB-hap versus GEL, implying that GELUKB-hap provides a powerful alternative imputation resource.

**Fig. 2:**
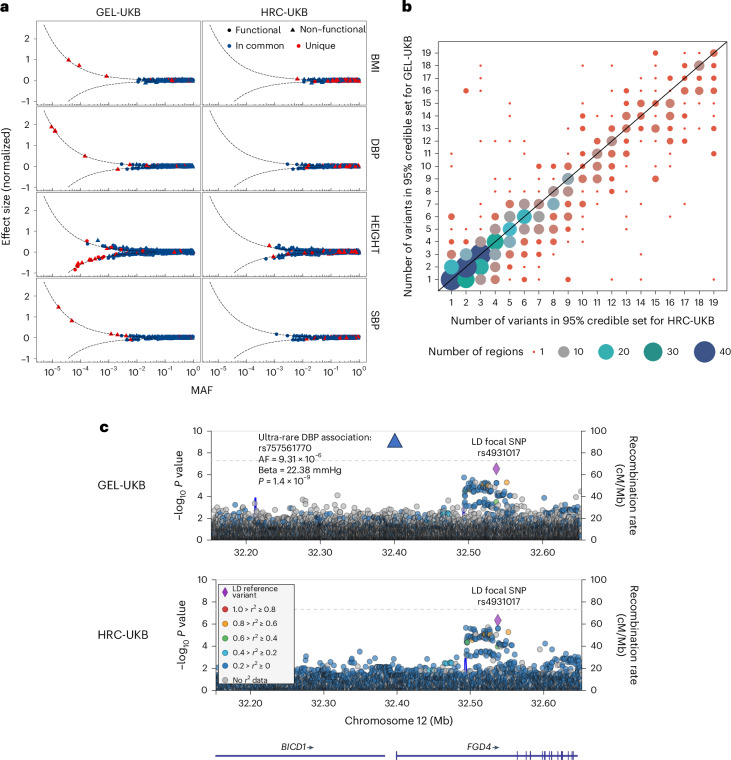
GEL-imputed UK Biobank data boost power to find common and rare associations. **a**, A set of independent genome-wide significant (*P* < 5 × 10^−8^) associations identified by step-wise regressions (conditioned joint analysis), and with INFO > 0.8, are plotted versus their imputed allele frequency (*x* axis). Blue points represent variants that were flagged by step-wise regressions in one dataset and also showed a significant GWAS association in the other dataset. Red points indicate variants unique to that dataset. The shape of the data points reflects the predicted consequences of the variants as determined by the Ensembl Variant Effect Predictor (release 105)^14^. Dots represent functional variants, including stop gained, stop lost, splice donor/acceptor, frameshift, in-frame insertion/deletion and missense variants and the triangles indicate non-functional variants. The dashed lines indicate the smallest hypothetical effect sizes that can be captured by the *P*-value threshold (*P* < 5 × 10^−8^) at power of 0.5. **b**, Comparison of the number of variants in the 95% credible sets for GEL-UKB and HRC-UKB fine-mapping results for standing height (capped at 20 variants; Methods). The circle sizes represent the number of fine-mapping regions showing each combination; plots below the diagonal correspond to GEL-UKB having fewer variants in the credible set compared to HRC-UKB. **c**, LocusZoom plot of ultra-rare-variant association (rs757561770) (in blue triangle) detected by GEL-UKB. The color indicates the linkage disequilibrium (LD) between SNPs and the focal SNP rs4931017, showing that rs757561770 is in low linkage disequilibrium with the focal SNP (*r*^2^ = 6.57 × 10^−6^). Blue lines show the regional recombination rate.

To demonstrate the use of GEL-UKB, we carried out exemplar GWAS on four quantitative traits: standing height, body mass index, systolic and diastolic blood pressure, with variant testing using REGENIE^7^. Across these traits, we found 31,699 and 30,711 significant (*P* < 5 × 10^−8^) rarer variant associations (MAF < 0.05) from GEL-UKB and HRC-UKB, respectively. The GEL-UKB imputed common variants also exhibited fewer likely false associations than HRC-UKB (Supplementary Note, Supplementary Table 2 and Supplementary Figs. 2–4). The resulting GEL-UKB GWAS *P* values generally show high correlation with those of TOPMed-UKB and UKB200K at sites they share (Supplementary Figs. 5 and 6). Compared to TOPMed-UKB, only GEL-UKB found ultra-rare associations (five at MAF < 10^−5^). The number of GEL-UKB-specific findings substantially exceeds those of TOPMed-UKB in all allele frequency bins (Supplementary Fig. 5), even common variants. We saw a useful improvement in fine-mapping (Methods) using GEL-UKB versus HRC-UKB; specifically, 44% of GEL-UKB based 95% credible sets contain fewer SNPs, while only 25% contain more SNPs (Fig. 2b and Supplementary Table 3).

**Extended Data Fig. 8:**
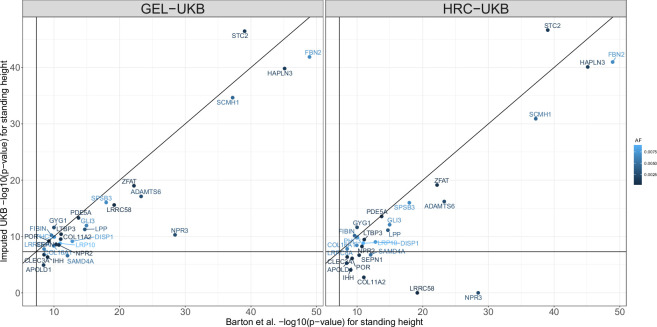
Comparing the *P*-values of GEL-UKB and HRC-UKB with rare likely causal coding variants identified by whole exome imputation. The *x*-axis shows the *P*-value using whole exome imputation^9^, and the *y*-axis shows the *P*-value using GEL-UKB (left) and HRC-UKB (right). The −log *P*-value is set to be 0 when the variant is not found. The dots are colors according to their allele frequency from the exome imputation data. The horizontal and vertical lines show the genome-wide significant threshold, that is 5 × 10^−8^.

A recent UKB exome sequencing-based association study reported 34 rarer (MAF < 0.05) GWAS hits across the four traits (*P* < 2.18 × 10^−11^) (ref. ^8^). At the same *P*-value threshold, we discovered 70% of these associations using GEL-UKB (76% at *P* < 5 × 10^−8^), compared to 56% using HRC-UKB (Supplementary Table 4). Comparing to the UKB whole-exome imputation GWAS results^9^, all but 4 of the 28 exome imputation likely causal rare coding variants associated with standing height (*P* < 5 × 10^−8^) were found to be significant using GEL-UKB versus all but 9 using HRC-UKB (Extended Data Fig. 8). Noticeably, our imputed data *P* values were more significant than those previously obtained using imputation from 150,000 sequenced UKB samples^10^ (Supplementary Table 7), perhaps due to the more powerful testing framework offered by REGENIE^7^ or improvements in GEL-based imputation.

At very rare variants (MAF < 5 × 10^−4^), several independent associations are discovered by GEL-UKB (Fig. 2a) but not HRC-UKB. For example, GEL-UKB identifies a new ultra-rare association signal for diastolic blood pressure at rs757561770 in *FGD4*, with allele frequency 9.31 × 10^−6^. Common variants in *FGD4* have previously been associated with hypertension^11^ (Fig. 2c). Notably, rs757561770 is intronic and shows no strong linkage disequilibrium (*r*^2^ > 0.7) with any identified coding variant (Supplementary Table 6). Because we test the entire genome, our results allow us to investigate whether similar large-effect variants (which in our example GWAS are only found at low frequency; Fig. 2b) occur in coding or non-coding DNA more generally. We identified 27 independent large-effect/rare-variant signals (MAF < 0.001; *P* < 5 × 10^−8^), across traits using step-wise regression (Methods). Among these, 15 are coding or splice site variants (*n* = 9) or in strong linkage disequilibrium (*r*^2^ > 0.7) with such a variant. Two more genic variants occur in 5′ untranslated regions (UTRs) (Supplementary Table 6). These 17 variants comprise 63% of all signals including, 16 of the 18 strongest associations by *P* value (Supplementary Table 6). If replicated for other phenotypes, this implies that it may be unusual for variation in non-coding regions, for example enhancers, to achieve dramatic trait effects—despite such regions dominating GWAS signals overall^12^. Because it seems likely that non-coding variants are able to strongly disrupt the binding of individual transcription factors, this might imply that (except in 5′ UTR regions), in most cases, no individual transcription factor binding site plays an essential functional role. Nonetheless, we still observed several cases implicating only non-genic sites—for example, two rare intronic signals for decreased height (rs773574844 and rs1414220739) near *SLC12A1*, a gene known to be associated with height and Bartter syndrome, whose symptoms include growth retardation^13^. We anticipate that, despite their modest effect sizes and limiting power at present (likely, even if genomes are fully sequenced), the number of non-coding associations will probably increase rapidly in the future once sample sizes become larger. Moreover, our results imply that imputation will be highly effective in identifying such associations, even for rare variants.

One unexpected finding for increased height was a tight ~1-kilobase (kb)-wide cluster of five independent low-frequency variants on chromosome 6 (Supplementary Table 7), including the rare missense variant rs957675208 (*HMGA1*/*LOC124901225*), in a region not reported by previous exome sequencing^8^ and exome imputation^9^ analyses or by HRC-UKB (low imputation INFO). Notably, rs957675208 shows the strongest height-increasing impact of any SNP in the whole genome, equivalent to gaining 3.5 cm of height. On further examination, three of the five variants are missense variants in *LOC124901225* and the remaining two variants are in the 5′ UTR of *HMGA1*, in a region not annotated in prior exome studies. It is unclear whether these associations reflect regulatory or direct coding roles. This gives one example of how the complete genome-wide coverage of the GEL-UKB data allows for more findings compared to previous approaches.

## Methods

This work was conducted under the approved UK Biobank applications numbered 48031 and 27960 and Genomics England Clinical Interpretation Partnership project ID RR91.

### Genomics England high-coverage sequencing data

The GEL 100,000 Genomes Project was launched in 2013, focusing on rare diseases and cancer. More than 120,000 genomes have been sequenced. It comprises genomes from 73,700 patients with rare diseases (disorders affecting ≤1 in 2,000 persons) and their close relatives and 46,539 genomes from patients with cancer^1^. The GEL reference panel described in this paper is built on the aggregated dataset (aggV2), comprising 78,195 samples from both rare disease and cancer germline genomes. Samples were sequenced with 150 bp (base pair) paired-end reads on the IlluminaHiSeq X platform and processed with the Illumina North Star Version 4 Whole Genome Sequenced Workflow (iSAAC Aligner v.03.16.02.19 and Starling small variant caller v.2.4.7) and aligned to the GRCh38 human reference genome. The individual gVCF files were aggregated into multisample VCF files using Illumina gVCF genotyper and normalized with vt v.0.57721. The aggregated multisample VCF dataset (aggV2) comprises over 722 million initial called SNPs and short indels (≤ 50 bp). Multi-allelic variants were decomposed into biallelic variants. The dataset includes 49,641 samples (63.48%) from individuals self-identifying as white British, 4,100 (5.24%) as ‘Other white’, 2,885 (3.69%) as Pakistani, 1,860 (2.3%) as Black, 1,751 (2.24%) as Indian and 12,277 samples (15.7%) as ‘Unknown’. According to the self-reported data, only 27,346 samples (34.97%) have no relatives in the reference panel; 11,584 (14.81%), 32,679 (41.79%) and 6,586 (8.43%) samples possess two, three and more than three family members in the dataset, respectively. We identified 12,816 (16.39%) samples as members of duo families and 35,106 (44.9%) as members of trio families, whereas 30,273 (38.71%) samples are treated as unrelated for phasing (Supplementary Note).

### Quality control

Before the quality control (QC) described here, sample-level QC was carried out by the GEL informatics team on variants called one sample at a time. We conducted further QC by pooling information across samples to remove false-positive sites. Specifically, we used aggregated VCFs, considering genotype quality, depth, missingness, allelic balance, Mendel errors, Hardy–Weinberg equilibrium and gnomAD^4^ allele frequency concordance. Because singletons observed in unrelated samples are difficult to phase accurately, these sites were removed. We applied two sets of QC rules. First, we applied a stringent rule set applied to all sites, including those de novo in GEL and very rare sites. Second, we applied a more lenient group of filters for relatively common sites (AF > 0.001) that also showed support from independent external datasets (TOPMed, HRC, 1000 Genomes and gnomAD) to avoid removing a proportion of genuine sites (for example, for a modest number of Mendel errors). For these sites, if they failed our stringent filters but passed with somewhat less stringent missingness, Mendel error and gnomAD frequency concordance thresholds, we included them, after separate phasing conditional on the phase of sites passing the more stringent thresholds, that is in a manner that did not impact the stringent sites. These sites were incorporated in the final dataset but with a QC flag indicating their slightly lower reliability. Overall, our filters reduced the initial number of sites from 722 million to 342 million (Supplementary Note and Supplementary Table 5).

### Phasing the GEL reference panel

We used a multistage phasing strategy leveraging the relatedness within GEL, in particular allowing phasing of singletons where possible.

1. We used the makeScaffold software (https://github.com/odelaneau/makeScaffold) to determine the phase of duo and trio samples (Supplementary Note) by direct transmission information (this phases most sites in these samples).
2. For remaining unphased genotypes in these related samples, with phases undetermined due to heterozygosity or missing data, phases were inferred using SHAPEIT4.2.2 (ref. ^15^), using the phased genotypes from step 1 as a scaffold.
3. To phase genotypes in the unrelated samples, we first phased the common variants (AF > 0.01) one chromosome at a time, using SHAPEIT4.2.2 and now using the genotypes (at these common sites) from step 1 and 2 in the related samples as a reference panel.
4. Finally, to phase the remaining sites: genotypes at rare variants in unrelated samples, we use SHAPEIT4.2.2 with the phased samples from steps 1 and 2 as a reference panel and the phased common variants from step 3 as a scaffold for these samples.
5. For sites only passing our lenient filters (‘Quality control’ section above and Supplementary Note), we used the results of step 4, for the sites on the UKB Axiom array sites passing the stringent filters, as a scaffold and then used SHAPEIT4.2.2 on the remaining genotypes.

Phasing for steps 1 and 3 was done at the entire chromosome level; for steps 2 and 4, it was carried out in regions of ~300,000 sites, with 30,000 sites on each side as buffer. The resulting phased regional segments were merged and concatenated using bcftools^16^. These phasing steps were computationally intensive and took ~6,500 CPU days in total to accomplish. The phased reference panel is stored in VCF format and has been made available for all GEL registered users on the GEL trusted research environment.

### Estimation of 1000 Genome trio phasing switch error rate

Phasing accuracy is important for direct biological interpretation of variants within GEL, as well as ensuring high-quality imputation in other samples and other downstream applications. We assessed the ability of the GEL panel to phase such external samples. Specifically, we phased the parents of mother–father–child trios included in the 1000 Genomes Project (but not HRC or GEL) using the reference panels from HRC and GEL. We then assessed the resulting phase accuracy by comparing phased haplotypes to those directly inferred using inheritance patterns to the child in each trio. The HRC reference panel was lifted over from the GRCh37 to the GRCh38 reference genome using GATK Picard LiftoverVCF^17^. The original GRCh37 HRC reference panel has 39,131,578 autosomal variants. We removed 13,813 variants either due to incompatibility between reference genomes or mismatching chromosome between the two reference genomes. The resulting autosomal GRCh38 HRC reference panel contains 39,115,765 variants and 27,165 samples. The 1000 Genome Project samples within the HRC reference panel were removed.

We analyzed only sites passing 1000 Genome Project data^18^ filters. The phasing test was carried out on 589 trio families from diverse ethnic backgrounds, using SHAPEIT4.2.2 (ref. ^15^). We tested all the heterozygous 1000 G sites for each individual reference panel, yielding a total of 1.04 × 10^9^ heterozygous sites (1.76 million per trio family) for the HRC panel and 1.16 × 10^9^ (1.9 million per trio family) for the GEL panel.

### Imputation testing of the 1000 Genomes Project samples

We used 2,405 samples from the 1000 Genomes Project to test the relative performance of imputation based on the GEL, TOPMed r2 and HRC imputation panels. We first performed quality control on the 1000 Genomes Project data by removing sites which either possess a missingness >5% or failed a Hardy–Weinberg equilibrium test, by having *P* < 10^−10^ in any of the 26 populations of the 1000 Genome Project. We then masked genotypes in 1000 Genomes Project sequencing samples, except the sites existing in the UK Biobank Axiom array, to mimic imputation using this array. This gave 716,473 biallelic SNPs across all autosomes. The pseudo-SNP array dataset was then phased one chromosome at a time using SHAPEIT4.1.2 (ref. ^15^). TOPMed imputation was carried out using the TOPMed imputation server with the TOPMed r2 reference panel and the imputation software minimac4 1.5.7 (ref. ^19^). IMPUTE5 (ref. ^20^) was used to impute from the GEL and HRC reference panels. We stratified imputation results into six groups: 661 AFR, 347 AMR, 504 EAS, 489 SAS, 313 non-Finnish European (NFE) samples and 91 GBR samples.

### UK Biobank imputation using the GEL reference panel

The UK Biobank SNP array data consist of 784,256 autosomal variants. We removed the set of 113,515 sites identified by the previous centralized UK Biobank analysis as failing quality control^6^ and an extra set of 39,165 sites failing a test of Hardy–Weinberg equilibrium on 409,703 GBR samples, with the *P*-value threshold of 10^−10^. The resulting UK Biobank SNP array data were mapped from the GRCh37 to GRCh38 genome build, using the GATK Picard LiftOver tool. Alleles with mismatching strand but matching alleles were flipped. We removed 495 sites because of incompatibility between the two reference genomes, resulting in a final SNP array incorporating 631,081 autosomal variants that we used for phasing and imputation. Haplotype estimation of the SNP array data is a prerequisite for imputation. Phasing was carried out one chromosome at a time using SHAPEIT4.2.2 without a reference panel, using the full set of UK Biobank samples. We ran SHAPEIT4 using its default 15 Markov chain Monte Carlo iterations and 30 threads. The runtime varied from 2 to 30 hours for each chromosome. Imputation of normal filter set and lenient filter set SNPs was carried out independently. Autosomal imputation using the GEL reference panel was performed using IMPUTE5 (v.1.1.4). The SNP array data were divided into 408 consecutive and overlapping chunks with ~5 megabases (Mb) for each chunk and 2.5 Mb buffer across the genome using the Chunker program in IMPUTE5 (ref. ^20^) and each chunk was further divided into 24 sample batches with each batch containing 20,349 samples. IMPUTE5 was run on each of the 9,792 subsets using a single thread and default settings, at a speed <4 min per genome, resulting in a total time of ~1,200 CPU days to impute all UK Biobank samples. The resulting imputed genotype dosages are stored in BGEN format and phasing information is stored in VCF format.

### Genome-wide association studies

We selected four quantitative traits to demonstrate the GWAS performance of the GEL-imputed UK Biobank data (GEL-UKB), compared to the HRCUK10K-imputed UKB (HRC-UKB) data on 429,460 GBR samples. These traits are standing height (HEIGHT), body mass index (BMI), systolic blood pressure (SBP) and diastolic blood pressure (DBP). Variants with minor allele count <5 are not included in testing. The phenotypes are transformed using rank inverse normal transformation (RINT) within sexes to ensure normally distributed input phenotypes and reduce the likelihood of false positives due to outliers. We also performed GWAS on the raw phenotype measures as a reference but, in our analyses, we use the RINT results if not otherwise specified. In addition, we followed the same procedure to perform GWAS using TOPMed imputed UKB (TOPMed-UKB) and 200,000 UKB sequencing data (UKB200K) on the UKB research analysis platform.

Samples between 40 and 70 years old are included and for each phenotype; outliers that are above ±4 s.d. from the mean value were removed^6^. SBP and DBP values are based on automated blood pressure readings, substituting in manual reading values when automated readings are not available. We calculated the mean SBP and DBP values from two automated (*n* = 418,755) or two manual (*n* = 25,888) blood pressure measurements. For individuals with one manual and one automated blood pressure measurement (*n* = 13,521), we used the mean of these two values. For individuals with only one available blood pressure measurement (*n* = 413), we used this single value. After calculating blood pressure values, we adjusted for blood pressure-lowering medication (*n* = 94,289) use by adding 15 and 10 mmHg to SBP and DBP, respectively^21^, for individuals on such medication.

GWAS effect size estimates and *P* values were obtained using REGENIE^7^. Throughout the paper, we present two-sided raw *P* values and use a widely used significance threshold of *P* < 5 × 10^−8^. We used the UKB SNP array data to estimate the LOCO predictors in REGENIE step 1 and the imputed data for step 2, accounting for sex, age, sex squared, sex × age and 20 principal components as covariates^7^. The association tests for GEL-imputed UKB (GEL-UKB) and HRCUK10K-imputed UKB (HRC-UKB) used the identical setup. The HRC-UKB summary statistics of the association tests were mapped using Picard LiftOver from GRCh37 to GRCh38 to compare the results with GEL-UKB. In all analyses, we used an INFO threshold of 0.3 for common imputed variants (MAF > 0.05) and 0.8 for rare imputed variants (MAF ≤ 0.05). Supplementary Fig. 1 shows that higher INFO thresholds are effective for detecting false-positive rare associations.

### Bayesian fine-mapping

Bayesian fine-mapping credible set size comparison was carried out on 1,660, 711, 505 and 546 non-overlapping regions for HEIGHT, BMI, SBP and DBP, respectively, on the basis of HRC-UKB GWAS summary statistics. These regions were defined by the following procedure. First, candidate regions were identified with width 0.125 cM plus 25 kb on each side of a significant marker. Overlapping candidate regions were successively merged until there were no remaining regions overlapping. We removed 60, 30, 33 and 51 regions for the above traits, respectively, in which GEL-UKB showed no significant sites (*P* < 5 × 10^−8^ in GWAS) for each trait. The recombination rate is based on the HapMap genetic map^22^. A detailed description of this approach can be found in refs. ^6,23^.

For each region, we assume a single causal variant—we call this model *M*. Given this, we define model *M*_*i*_ to be the model where SNP *i* is the causal variant. We seek the probability of *M*_*i*_ given the data and that model *M* is true. This posterior Pr(*M*_*i*_|**X**, *M*) can be written in terms of the Bayes factor relating the probability of the data given *M*_*i*_ versus the probability of the data under the null model with no associated SNP in the region, BF_*i*_. Further, BF_*i*_ can be approximated by an asymptotic Bayesian factor (ABF_*i*_):

$$\Pr \left({M}_{i}|{\mathbf{X}},M\right)=\frac {{\rm{BF}}_{{i}}}{\mathop{\sum }\limits_{i=1}^{k}{\rm{BF}}_{{i}}}\approx \frac{{\rm{ABF}}_{i}}{\mathop{\sum }\limits_{i=1}^{k}{\rm{ABF}}_{i}}.$$

ABF_*i*_ can be calculated using the standard error (*V*_*i*_) and *Z* score (*z*) estimated by REGENIE^6^. In each region, the smallest possible 95% credible set of potential causal markers can be obtained by successively including the sites with the highest probabilities, to accumulatively reach 0.95. Model *M* requires a prior (a Gamma distribution) on effect sizes; we choose this prior *W* to have parameters 0.2^2^ and 0.02^2^ but found that the results are not particularly sensitive to the choice of the prior.

### Conditional joint analysis using step-wise regression

A standard GWAS uses a marginal model considering one variant at a time, while a joint model considers all the selected variants and estimates their joint effect simultaneously to remove rare-variant signals that are explained by stronger signals at more common nearby SNPs^8^. We performed a conditional joint analysis via a step-wise forward selection procedure, considering each chromosome separately. First, we defined the set **S** of genome-wide significant variants in one chromosome (*P* < 5 × 10^−8^) in the marginal regression using REGENIE. We initialized a set of variants **R** as the most significant variant in the marginal regression. Given the current value of **R**, we calculate the *P* value of all the remaining variants in **S** one at a time, conditioned on **R** and the covariates used for the initial GWAS. We then move the variant with the smallest conditional *P* value from **S** to **R**, until this smallest *P* value is no longer genome-wide significant. This approach identifies a set of variants that are independently significant and account for all the genome-wide association signals (note that this set is not unique), while also accounting for linkage disequilibrium between sites. To identify rare causal variants within UKB found using GEL-UKB imputation, we considered only those variants found by this step-wise forward selection approach. The full conditional joint analysis results can be found in Supplementary Table 7.

### Reporting summary

Further information on research design is available in the Nature Portfolio Reporting Summary linked to this article.

## Online content

Any methods, additional references, Nature Portfolio reporting summaries, source data, extended data, supplementary information, acknowledgements, peer review information; details of author contributions and competing interests; and statements of data and code availability are available at 10.1038/s41588-024-01868-7.

## Supplementary information

Supplementary Information Supplementary Notes 1–4, Tables 1–5 and Figs. 1–6.

Reporting Summary

Supplementary Table 6 GWAS summary statistics of rare step-wise regression significant sites (AF < 0.001).

Supplementary Table 7 GWAS summary statistics of all step-wise regression significant sites.

## Data Availability

The GEL haplotype reference panel is available in the GEL Research Environment (https://re-docs.genomicsengland.co.uk/ox_aggv2/) to approved researchers in the Genomics England Research Network (https://www.genomicsengland.co.uk/research/academic/join-research-network). The UK Biobank data imputed using the GEL haplotype reference panel are available to those with approved access to the UK Biobank resource and described on the UK Biobank showcase (https://biobank.ctsu.ox.ac.uk/crystal/field.cgi?id=21008). The GWAS summary statistics can be downloaded from GWAS Catalog under the study accession codes from GCST90435412 to GCST90435415.

## Code availability

All analyses were performed using previously published or developed tools, as indicated in the Methods. SHAPEIT4 (v.4.1.2) was used to phase the GEL reference panel and the phasing experiment of 1000 Genomes samples (https://odelaneau.github.io/shapeit4/). The imputation of UK Biobank samples was carried out by IMPUTE5 (v.1.1.4), which is freely available for academic use (https://jmarchini.org/software/). The imputation experiment using TOPMed reference panel was carried out on the TOPMed imputation server (https://imputation.biodatacatalyst.nhlbi.nih.gov/). REGENIE was used to perform GWAS (https://rgcgithub.github.io/regenie/). The following open source software was used for the data processing and quality control pipeline: BCFTools (https://samtools.github.io/bcftools/), GATK Picard LiftoverVCF (https://gatk.broadinstitute.org/hc/en-us/articles/360037060932-LiftoverVcf-Picard). No custom code was developed or used.
