# Supplementary material for: A Genomics England haplotype reference panel and imputation of UK Biobank
Source: Nat Genet. 2024 Aug 12;56(9):1800–3. doi: 10.1038/s41588-024-01868-7 (PMC11387190; doi:10.1038/s41588-024-01868-7)
Supplement: Supplementary file 2 — Reporting Summary [file 41588_2024_1868_MOESM2_ESM.pdf]

nature portfolio

Corresponding author(s): Sinan Shi and Simon Myers

Last updated by author(s): 1/7/2024

Reporting Summary

Nature Portfolio wishes to improve the reproducibility of the work that we publish. This form provides structure for consistency and transparency in reporting. For further information on Nature Portfolio policies, see our Editorial Policies and the Editorial Policy Checklist.

Statistics

For all statistical analyses, confirm that the following items are present in the figure legend, table legend, main text, or Methods section.

n/a	Confirmed	
☐	☒	The exact sample size (*n*) for each experimental group/condition, given as a discrete number and unit of measurement
☒	☐	A statement on whether measurements were taken from distinct samples or whether the same sample was measured repeatedly
☐	☒	The statistical test(s) used AND whether they are one- or two-sided<br>*Only common tests should be described solely by name; describe more complex techniques in the Methods section.*
☐	☒	A description of all covariates tested
☐	☒	A description of any assumptions or corrections, such as tests of normality and adjustment for multiple comparisons
☐	☒	A full description of the statistical parameters including central tendency (e.g. means) or other basic estimates (e.g. regression coefficient) AND variation (e.g. standard deviation) or associated estimates of uncertainty (e.g. confidence intervals)
☐	☒	For null hypothesis testing, the test statistic (e.g. *F*, *t*, *r*) with confidence intervals, effect sizes, degrees of freedom and *P* value noted<br>*Give P values as exact values whenever suitable.*
☐	☒	For Bayesian analysis, information on the choice of priors and Markov chain Monte Carlo settings
☒	☐	For hierarchical and complex designs, identification of the appropriate level for tests and full reporting of outcomes
☒	☐	Estimates of effect sizes (e.g. Cohen's *d*, Pearson's *r*), indicating how they were calculated

Our web collection on statistics for biologists contains articles on many of the points above.

Software and code

Policy information about availability of computer code

Data collection	There is no data collection process in this study.
Data analysis	SHAPEIT4 (v4.1.2) is used to phase the GEL reference panel and the phasing experiment of 1000 Genomes samples (https://odelaneau.github.io/shapeit4/). The imputation of UK Biobank samples is carried out by IMPUTE5 (v1.1.4), which is freely available for academic use (https://jmarchini.org/software/). The imputation experiment using TOPMed reference panel is carried out on the TOPMed imputation server (https://imputation.biodatacatalyst.nhlbi.nih.gov/). REGENIE is used to perform genome-wide association studies (https://rgcgithub.github.io/regenie/). The following open source software were used for the data processing and quality control pipeline, BCFTools (https://samtools.github.io/bcftools/), GATK Picard LiftoverVCF (https://gatk.broadinstitute.org/hc/en-us/articles/360037060932-LiftoverVcf-Picard).

For manuscripts utilizing custom algorithms or software that are central to the research but not yet described in published literature, software must be made available to editors and reviewers. We strongly encourage code deposition in a community repository (e.g. GitHub). See the Nature Portfolio guidelines for submitting code & software for further information.

Data

Policy information about availability of data

All manuscripts must include a data availability statement. This statement should provide the following information, where applicable:

- Accession codes, unique identifiers, or web links for publicly available datasets
- A description of any restrictions on data availability
- For clinical datasets or third party data, please ensure that the statement adheres to our policy

The GEL haplotype reference panel is available within the GEL Research Environment (https://re-docs.genomicsengland.co.uk/ox_aggv2/) to approved researchers of Genomics England Research Network (https://www.genomicsengland.co.uk/research/academic/join-research-network). The imputed UK Biobank data imputed using the GEL haplotype reference panel is available to those with approved access to the UK Biobank resource and described on the UK Biobank showcase here https://biobank.ctsu.ox.ac.uk/crystal/field.cgi?id=21008

Research involving human participants, their data, or biological material

Policy information about studies with human participants or human data. See also policy information about sex, gender (identity/presentation), and sexual orientation and race, ethnicity and racism.

Reporting on sex and gender	No sex or gender analyses were conducted in the study. Biological sex of participants (provided by UK Biobank) is used as covariate for GWAS.
Reporting on race, ethnicity, or other socially relevant groupings	We used the population labels defined by 1000 Genomes and UK Biobank. The imputation accuracy is largely affected by genetic distances between the reference populations and the target populations. In the imputation experiment, we stratified the 1000 Genomes samples using the population labels defined by 1000 Genomes to demonstrate the GEL panel has better overall imputation performance for British samples, since the samples collected in our reference panel are more similar to that of the British samples.
Population characteristics	N/A
Recruitment	N/A
Ethics oversight	This study has been approved by Genomics England GeCIP RR91 and UK Biobank application 48031 and 27960.

Note that full information on the approval of the study protocol must also be provided in the manuscript.

Field-specific reporting

Please select the one below that is the best fit for your research. If you are not sure, read the appropriate sections before making your selection.

☒ Life sciences ☐ Behavioural & social sciences ☐ Ecological, evolutionary & environmental sciences

For a reference copy of the document with all sections, see nature.com/documents/nr-reporting-summary-flat.pdf

Life sciences study design

All studies must disclose on these points even when the disclosure is negative.

Sample size	We used 78,195 Genomics England samples to build the reference panel and 488,315 UK Biobank samples to create the UKB imputed data.
Data exclusions	No specific steps were performed for data exclusion, but we excluded samples who have withdrawn from the UKB from our study.
Replication	We validated our GWAS findings using GEL-imputed UKB data through comparing the results to HRC+UK10K imputed (Bycroft et al. 2018), TOPMed imputed (Taliun et al., 2021) UKB data and 200K WGS UKB data. The resulting p-values of variants in common show high correlation and improved power for finding rare associations (Supplementary Fig. 5-6). However, a precise replication of GWAS findings is not possible (in common with many UKB studies), due to the difference in sample sizes and imputation accuracies.<br><br>Because our paper is about imputation and testing, we believe this validates the main claims in the paper. For testing of phasing and imputation accuracy, we were able to replicate our findings across many individuals (from the 1000G), chromosomes, and populations.
Randomization	This is not relevant to our study, because we did not select the study participants and we do not examine any treatment or experimental intervention.
Blinding	The identities of study participants are anonymous to us, for both GEL and UKB; because we did not examine any specific treatment, this question is not otherwise relevant.

Reporting for specific materials, systems and methods

We require information from authors about some types of materials, experimental systems and methods used in many studies. Here, indicate whether each material, system or method listed is relevant to your study. If you are not sure if a list item applies to your research, read the appropriate section before selecting a response.

Materials & experimental systems

n/a	Involved in the study
☒	☐ Antibodies
☒	☐ Eukaryotic cell lines
☒	☐ Palaeontology and archaeology
☒	☐ Animals and other organisms
☒	☐ Clinical data
☒	☐ Dual use research of concern
☒	☐ Plants

Methods

n/a	Involved in the study
☒	☐ ChIP-seq
☒	☐ Flow cytometry
☒	☐ MRI-based neuroimaging

Plants

Seed stocks

Report on the source of all seed stocks or other plant material used. If applicable, state the seed stock centre and catalogue number. If plant specimens were collected from the field, describe the collection location, date and sampling procedures.

Novel plant genotypes

Describe the methods by which all novel plant genotypes were produced. This includes those generated by transgenic approaches, gene editing, chemical/radiation-based mutagenesis and hybridization. For transgenic lines, describe the transformation method, the number of independent lines analyzed and the generation upon which experiments were performed. For gene-edited lines, describe the editor used, the endogenous sequence targeted for editing, the targeting guide RNA sequence (if applicable) and how the editor was applied.

Authentication

Describe any authentication procedures for each seed stock used or novel genotype generated. Describe any experiments used to assess the effect of a mutation and, where applicable, how potential secondary effects (e.g. second site T-DNA insertions, mosiacism, off-target gene editing) were examined.
